# Infrared surface plasmons on a Au waveguide electrode open new redox channels associated with the transfer of energetic carriers

**DOI:** 10.1126/sciadv.abm9303

**Published:** 2022-05-18

**Authors:** Zohreh Hirbodvash, Oleksiy Krupin, Howard Northfield, Anthony Olivieri, Elena A. Baranova, Pierre Berini

**Affiliations:** 1 Department of Physics, University of Ottawa, 150 Louis Pasteur, Ottawa, ON K1N 6N5, Canada.; 2 Center for Research in Photonics, University of Ottawa, 25 Templeton St., Ottawa, ON K1N 6N5, Canada.; 3 Department of Chemical and Biological Engineering, University of Ottawa, 161 Louis-Pasteur, Ottawa, ON K1N 6N5, Canada.; 4 Centre for Catalysis Research and Innovation, University of Ottawa, 161 Louis-Pasteur, Ottawa, ON K1N 6N5, Canada.; 5 School of Electrical Engineering and Computer Science, University of Ottawa, 800 King Edward Ave., Ottawa, ON K1N 6N5, Canada.

## Abstract

Plasmonic catalysis holds promise for opening new reaction pathways inaccessible thermally or for improving the efficiency of chemical processes. We report a gold stripe waveguide along which infrared (λ_0_ ~ 1350 nanometers) surface plasmon polaritons (SPPs) propagate, operating simultaneously as an electrochemical working electrode. Cyclic voltammograms obtained under SPP excitation enable oxidative processes involving energetic holes to be investigated separately from reductive processes involving energetic electrons. Under SPP excitation, redox currents increase by 10×, redox potentials decrease by ~2× and split in correlation with photon energy, and the charge transfer resistance drops by ~2× as measured using electrochemical impedance spectroscopy. The temperature of the working electrode was monitored in situ, ruling out thermal effects. Chronoamperometry measurements with SPPs modulated at 600 hertz yield a commensurately modulated current response, ruling out thermally enhanced mass transport. Our observations indicate opening of optically controlled nonequilibrium redox channels associated with energetic carrier transfer to the redox species.

## Teaser

Infrared surface plasmons open nonequilibrium redox channels associated with energetic electron and hole transfer.

## INTRODUCTION

Surface plasmon polaritons (SPPs) on metal surfaces have useful properties, such as strong field enhancement and subwavelength localization (*1*), which have long-driven vigorous interest in these waves. SPPs are damped by absorption in the metal (*2*), which limits their propagation length and lifetime. However, the absorption of SPPs creates energetic carriers (cf. hot electrons and holes) along the surface of the metal (*3*), which can be exploited in device applications or in catalysis (*4*–*7*), thereby turning what is often viewed as a drawback into a benefit. This route for creating energetic carriers is particularly compelling, given the high efficiency with which SPPs can be excited optically. These attributes drive research on plasmonic catalysis, motivated by a desire to open reaction pathways that are inaccessible thermally or to improve the efficiency of chemical processes by involving energetic carriers.

A metal surface supporting SPPs can double as a working electrode (WE) within an electrochemical cell. These electrodes have been constructed as a metal film on a prism in the Kretschmann configuration (*8*, *9*) or as metal nanoparticles on a conductive substrate (*10*, *11*). Redox reactions occurring on the surface of an electrode can be probed using SPPs localized thereon, revealing subtle details of reactions. For instance, SPPs were used to image the local current density directly on a planar WE (*8*). Alternatively, the optical performance of plasmonic structures can be tuned electrochemically, e.g., by shifting the SPP resonance of Au nanorods via charge injection (*11*). Electrochemical cells that incorporate plasmonic structures are also of interest as multimodal chemical transducers (*9*, *12*).

Recently, attention has turned to investigating the role played by energetic carriers created by SPP absorption in electrochemical reactions (*13*). Advantageously, redox currents are easily measured, proportional to reaction rates, and directly connected to experimental conditions, including plasmonic effects. Furthermore, processes involving energetic holes can be separated from processes involving energetic electrons by investigating oxidation or reduction reactions separately.

The creation of energetic carriers via SPP absorption invariably causes the temperature of the metal to rise, with heat diffusing into the nearby reaction volume. Given that chemical reactions are temperature dependent, separating the roles of temperature and energetic carriers is not trivial but essential to proper interpretation of results (*14*–*18*).

Empirically and in general, a reaction rate *K* increases exponentially with temperature following the Arrhenius law (*15*, *16*, *18*): *K* = *A*·exp[−*E*_a_/(*R*(*T*_0_ + *cI*))], where *E*_a_ is the molar activation energy, *R* is the gas constant, *T*_0_ is the nominal temperature, *c* is a photothermal conversion coefficient, and *A* is a constant depending on the reaction. It is generally a good assumption that the temperature of a plasmonic structure increases linearly with incident intensity (*T* ∝ *cI*) as long as the optical response remains linear. Conversely, the reaction rate should depend linearly on optical intensity if energetic carriers play a role because their number is proportional to the number of SPP quanta absorbed, which depends on the number of incident photons (intensity). In principle, the roles of temperature and energetic carriers can be distinguished by varying the intensity and observing whether the reaction rate evolves exponentially or linearly (respectively). However, this approach has pitfalls. For instance, an exponential appears linear for small changes in argument (cf. Taylor series), so experiments involving small intensity changes could imply an incorrect trend.

In electrochemical systems, increasing electrode and electrolyte temperature affects the equilibrium potential and electron transfer rates to/from the redox species and leads to convection that works to cool the system and enhance mass transport to the electrode (*19*–*23*). Thermal modeling, including diffusion, convection, and mass transport (*21*), predicts an approximately linear increase in redox currents with heating power (*i* ∝ *P^a^*, *a* = 0.8 to 1) rather than exponential (as might be expected from the Arrhenius law), and a current rise time of at least 10 s upon onset of heating. Trends with temperature are not straightforward, so electrochemical cells should be thermally stabilized, the electrode temperature should be monitored, independent thermal control experiments carried out, and optical variables in addition to intensity should be varied to separate the role of temperature from that of energetic carriers.

Much of the work carried out to date in plasmonic catalysis involved colloidal arrangements of Au nanoparticles illuminated at visible wavelengths (e.g., λ_0_ = 532 nm, *h*υ = 2.33 eV) (*4*–*7*, *13*). This scenario, although convenient, poses challenges. For instance, the temperature in the immediate vicinity of nanoparticles can be difficult to predict and measure because of collective effects (*16*). In addition, carriers excited in Au at wavelengths above the interband threshold (*h*υ ~ 2 eV) have very short lifetimes (because of electron-electron scattering at high carrier energies) (*24*).

High energy carriers (*h*υ > 2 eV) are generally deemed essential to catalyze reactions via SPPs. Contrary to this broadly held view, we use here lower-energy infrared photons (λ_0_ ~ 1350 nm, *hυ* ~ 1 eV) to excite SPPs and energetic carriers in Au. Under this excitation, the carriers have energies at most 1 eV above *E*_F_, and longer lifetimes, or longer attenuation lengths (*L*_e_ ~ 74 nm, *L*_h_ ~ 55 nm) (*25*), as the main carrier damping mechanism is electron-phonon scattering. Furthermore, we use a thin Au stripe as an SPP waveguide and WE, which offers several advantages over colloidal nanoparticles: The WE is defined lithographically and is well understood structurally. The temperature of the WE under SPP excitation is determined in situ using calibrated resistance measurements. SPPs propagate over the entire length of the WE with exclusive and complete overlap. Last, the thickness of the WE (*t*) is less than the excited carrier attenuation lengths in Au (*L*_e_, *L*_h_), enabling multiple internal carrier reflections that enhance their escape probability (*26*).

## RESULTS

**Fig. 1.**
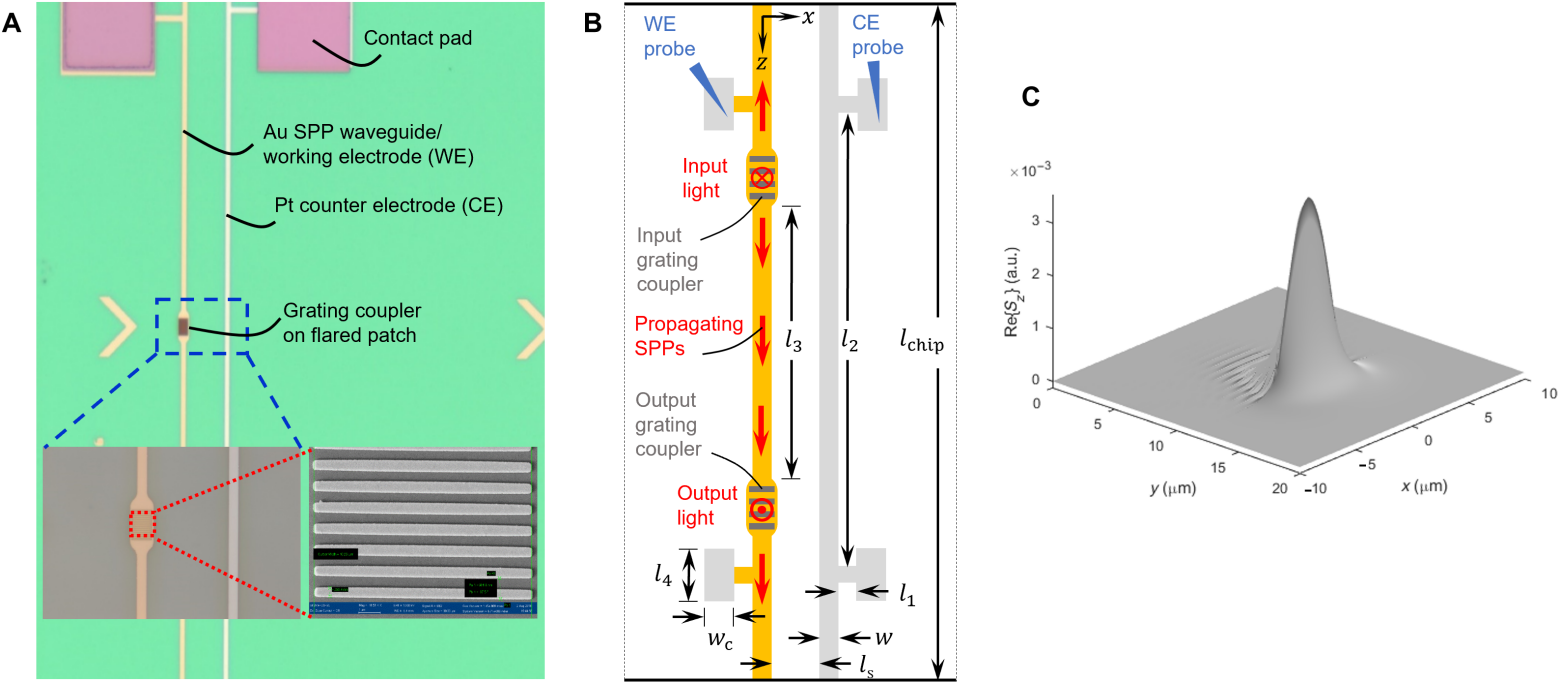
Plasmonic waveguide and electrodes. (**A**) Microscope image in top view of a chip bearing Au SPP waveguides/WEs, Pt CEs, Au grating couplers, and electrical contact pads on a multilayer substrate (appears green under bright-field optical microscopy). The bottom insets show scanning electron microscope (SEM) images of a grating coupler. (**B**) Geometry of the electrodes in top view: *l*_1_ = 29 μm, *l*_2_ = 2600 μm, *l*_3_ = 1850 μm, *l*_4_ = 250 μm, *l*_chip_ = 3000 μm, *w*_c_ = 100 μm, *w* = 5 μm, and *l*_s_ = 40 μm. The thickness of both electrodes (along *y*) is *t* = 35 nm. Experimental scheme: The WE and CE are contacted using external probes to form a three-electrode electrochemical system with an Ag/AgCl reference electrode. Input laser light, normally incident on the input grating coupler, excites SPPs that propagate along the WE. The output grating coupler converts the SPPs to output light emerging normally from the chip, which is captured and measured. (**C**). Computed distribution of Re{*S_z_*}, where *S_z_* is the component of the Poynting vector (proportional to mode intensity) in the direction of propagation (along *z*). a.u., arbitrary units.

Metallic structures were fabricated on a multilayer substrate, as shown in Fig. 1, using standard nanofabrication techniques (*27*, *28*). Figure 1A gives a microscope image of a Au stripe designed to operate simultaneously as an SPP waveguide and WE. The image also shows a nearby Pt stripe used as a counter electrode (CE) and thick contact pads (>200 nm) for electrical probing. The chip is immersed in a petri dish filled with the redox species in electrolyte in which a Ag/AgCl reference electrode is dipped, thereby forming a three-electrode electrochemical cell. Glycerol was added to the electrolyte to adjust its refractive index (*n* = 1.3325, λ_0_ = 1312 nm), as the solution also acts as the upper cladding of the SPP waveguide. The petri dish was mounted on a thermoelectric cooler (TEC) driven by a temperature controller using an electronic thermometer dipped in the electrolyte for feedback control. Unless stated otherwise, the bulk electrolyte temperature was maintained constant to 20°C during all experiments. (cf. Materials and Methods and fig. S1).

Figure 1B illustrates the geometry of the electrodes, including their dimensions. The design of the Pt stripe (CE) is identical to that of the Au stripe (WE). Both are *t* = 35 nm thick (<*L*_e_, *L*_h_ in Au) and were deposited on 0.3 nm of Ti as an adhesion layer.

The geometry of the WE is constrained by its dual use as an SPP waveguide. The substrate is a multilayer stack, consisting of 15 periods of alternating layers of SiO_2_/Ta_2_O_5_, as a truncated one-dimensional photonic crystal, on a Si wafer. Bloch long-range SPPs (*27*–*29*) propagate over the full length of the Au stripe, with a field distribution that surrounds the stripe—this is a key attribute of the structure as SPPs overlap exclusively and completely the WE. Figure 1C shows the distribution of Re{*S_z_*} of this mode, where *S_z_* is the component of the Poynting vector in the direction of propagation (*z*) and is proportional to the mode intensity. The computation was carried out using the method of lines, as described under Materials and Methods.

The bottom insets in Fig. 1A show scanning electron microscope (SEM) images of a grating coupler, as used for optical input and output coupling to Bloch long-range SPPs using perpendicular optical fibers (*27*–*29*). The experimental scheme is illustrated in Fig. 1B (cf. Materials and Methods and fig. S1). Advantageously, the arrangement in transmission enables optimization in situ of both optical alignments.

Once optical alignments were established, the laser wavelength was swept over the range of 1300 to 1370 nm, while the output optical power was monitored (fig. S2). The output power was maximum over the 1330- to 1370-nm range, which agrees well with the design wavelength of the grating couplers and waveguide (*28*, *29*). Three operating wavelengths were used for the experiments: 1330, 1350, and 1370 nm.

All experiments were carried out with 0.5 mM K_3_[Fe(CN)_6_] + 100 mM KNO_3_ electrolyte, using a triangular potential waveform at a scan rate of 100 mV/s. Applying such a potential to the system leads to the chemically reversible reaction of potassium ferricyanide {K_3_[Fe(CN)_6_]} to potassium ferrocyanide {K_4_[Fe(CN)_6_]} as a redox couple in a one-electron transfer process

$$K_{4}[\text{Fe}{\left(\text{CN}\right)}_{6}]↔K_{3}[\text{Fe}{\left(\text{CN}\right)}_{6}]+e^{-}$$

**Fig. 2.**
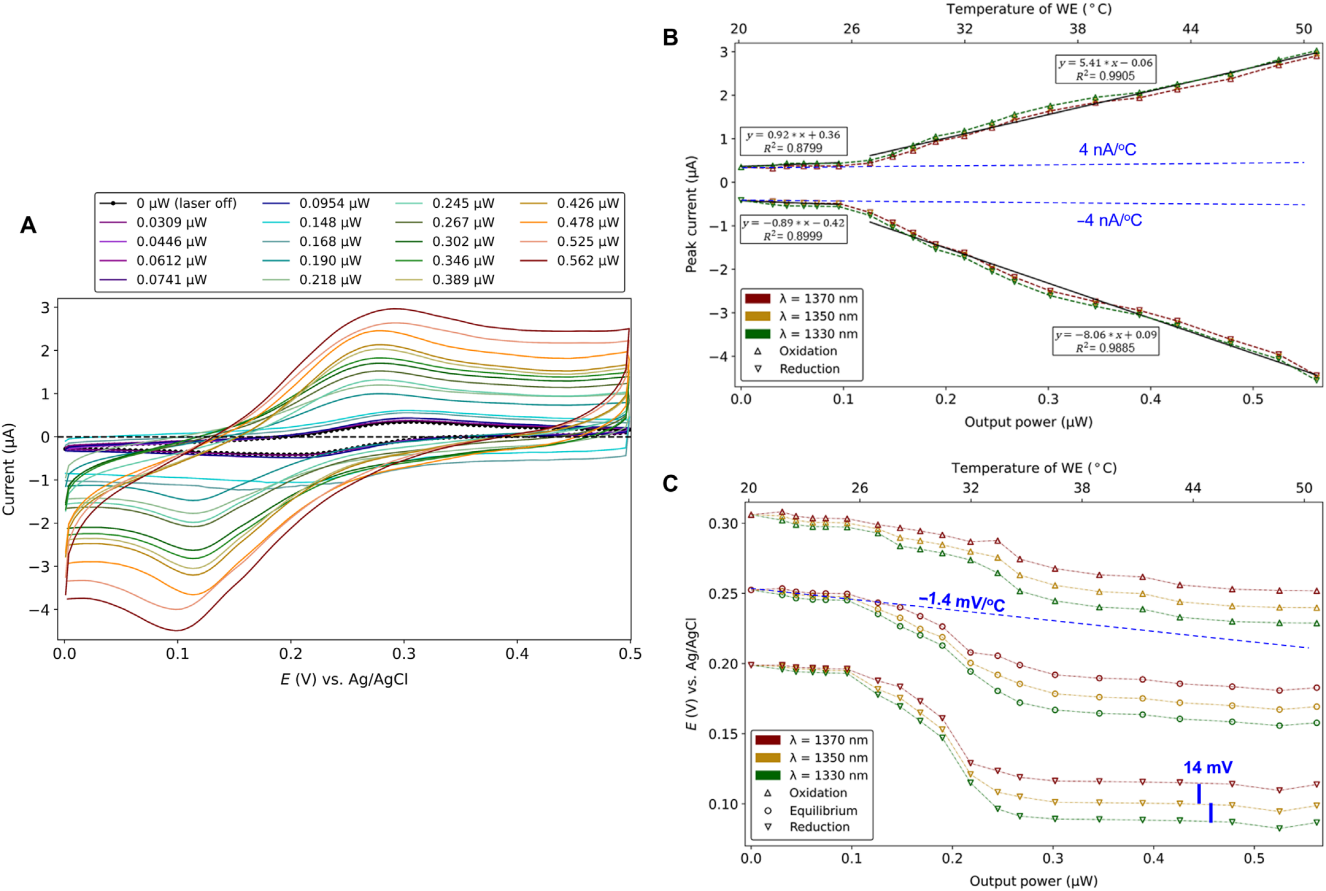
CV under optical illumination. (**A**) CV curves obtained on a Au WE, in 0.5 mM K_3_[Fe(CN)_6_] + 100 mM KNO_3_ electrolyte, at a scan rate of 100 mV/s, for increasing output optical power (legend) at λ_0_ = 1350 nm. The incident optical power ranged from 0 to 6.3 mW. The reference CV curve (laser off) is plotted in black dots. (**B**) Redox current peaks, and (**C**) potentials versus output optical power, from CV curves measured at λ_0_ = 1330, 1350, and 1370 nm. Linear models of the peak redox currents at λ_0_ = 1350 nm are plotted as the solid black lines in (B) (slopes have units of amperes per watt). The solid vertical blue bars in (C) measure 14 mV, corresponding to the approximate photon energy separating the three optical wavelengths (~14 meV). Linear thermal trends, measured independently, are added as the blue dashed lines to (B) and (C).

Cyclic voltammetry (CV) was carried out on a Au WE, without optical illumination, providing a reference CV curve. CV was then carried out under the same conditions but with SPPs propagating along the Au WE as a function of incident optical power (intensity) and wavelength. The output optical power was monitored during all experiments. The measured CV curves, shown in Fig. 2A for λ_0_ = 1350 nm, change markedly as the optical power increases. The CV curves for λ_0_ = 1330 and 1370 nm show similar changes (fig. S3).

The current increases significantly with optical power compared to the reference case (no illumination). Figure 2B plots the peak oxidation and reduction currents versus the output optical power for the three wavelengths of interest, revealing an increase of ~10× in both over the power range investigated. The reduction current increases more than the oxidation current. A clear threshold is observed in Fig. 2B at an output power of ~0.1 μW, about which different linear trends are evident. Linear models of peak current versus output optical power, fitted for each segment at λ_0_ = 1350 nm, are plotted as the solid black lines and given in the corresponding legends.

The potentials corresponding to the peak redox currents also change significantly with optical power, as shown in Fig. 2C. The reduction potential decreases by ~2×, the oxidation potential by ~1.3×, and the equilibrium potential (mean of the redox potentials) by ~1.7× relative to the Ag/AgCl reference electrode. Again, a clear threshold is observed at an output power of ~0.1 μW. Beyond threshold, the redox potentials decrease commensurately with increasing photon energy: Δ(*h*υ) = 13.8 meV separates λ_0_ = 1330 from 1350 nm, and Δ(*h*υ) = 13.4 meV separates λ_0_ = 1350 from 1370 nm; vertical blue bars of 14 mV on Fig. 2C illustrate this point.

The resistance of the WE was measured in situ as a function of optical power and compared to calibrated resistances to determine its temperature (fig. S4). The temperature of the WE, added to Fig. 2 (B and C) as the top horizontal scale, spans about 30°C.

Various control experiments were carried out in situ under identical experimental conditions, as described in the text that follows. Control experiments without the redox species produced featureless and noisy CV curves whether the illumination was on or off (fig. S5).

Control experiments with the laser on while misaligning the input optical fiber (in various ways) were carried out. The CV curves would always return to the reference case (no illumination) as soon as coupling to the input grating was lost, confirming that the excitation of SPPs on the WE was essential to the changes observed in Fig. 2.

Temperature control experiments without illumination were carried out using the TEC placed under the petri dish to cool then heat the entire cell (electrodes and electrolyte) in a uniform and controlled manner. CV curves obtained at different temperatures, ranging from 10° to 40°C, show changes in peak redox currents of about 30%, following linear trends with temperature of fitted slopes ±4 nA/°C, consistent with thermally induced mass-transport effects (fig. S6) (*21*). The reduction and oxidation potentials change by 10 to 20%. The temperature dependence of the equilibrium potential was fit to a linear model yielding a slope of −1.4 mV/°C, in good agreement with thermally induced shifts reported in the literature for the ferricyanide/ferrocyanide system (*19*, *30*–*32*). Linear thermal trends based on these measurements were added to Fig. 2 (B and C) as the dashed blue lines.

Further temperature control experiments without illumination were carried out by directly heating the WE resistively through current injection while maintaining the bulk electrolyte temperature to 20°C (cf. Materials and Methods and fig. S7). By resistively heating the WE, a thermal gradient is produced in the electrolyte, resembling closely the heat gradient produced by absorption of the propagating SPPs. CV curves obtained while directly heating the WE to temperatures ranging from 20° to 50°C (fig. S8) are very similar to those obtained by heating the entire cell in a uniform manner (fig. S6), including the linear thermal trends for the peak redox currents and the equilibrium potential (legends). Thus, small thermal gradients in our system, generated resistively or optically, do not have a significant impact on the results.

**Fig. 3.**
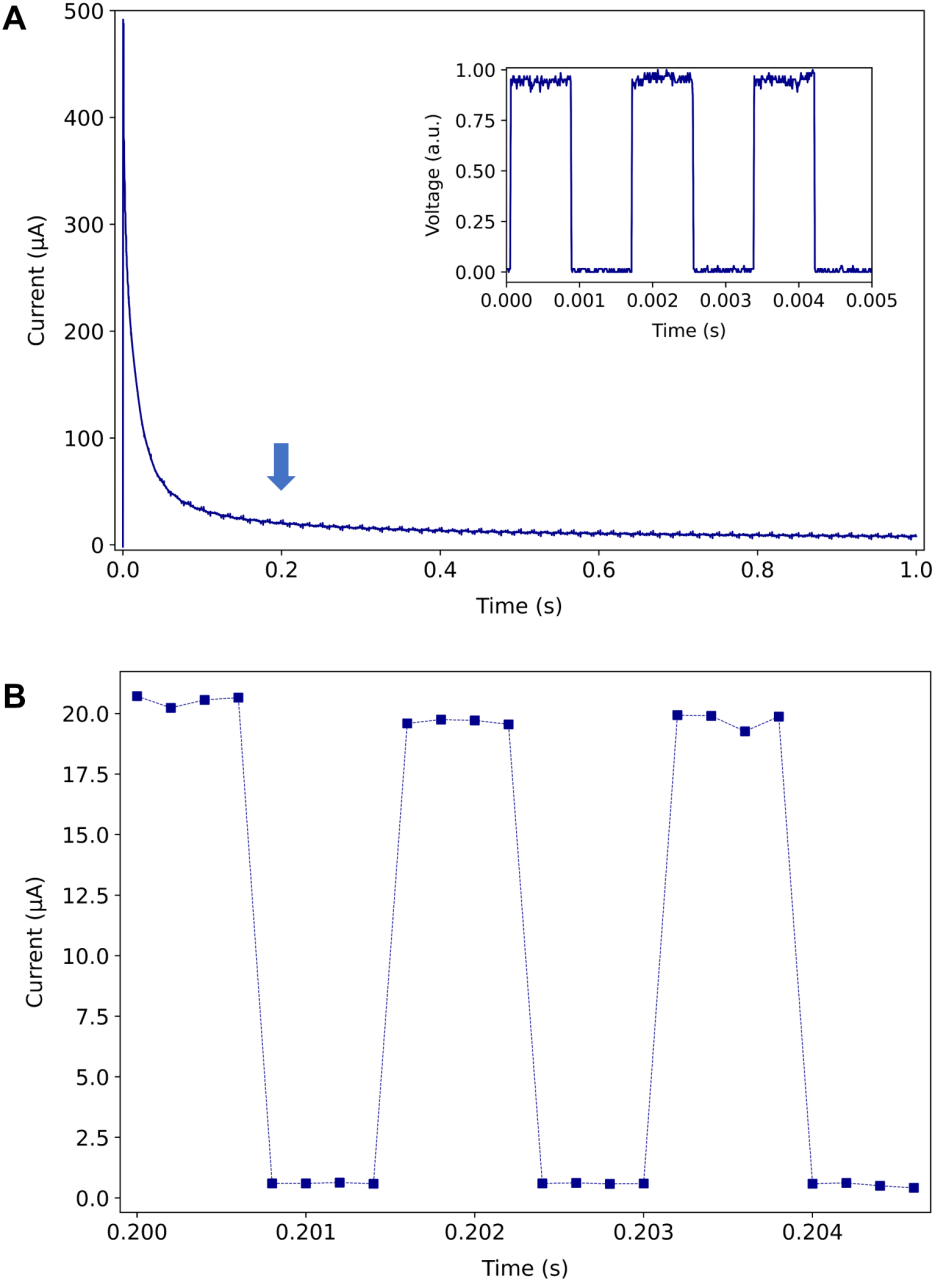
Chronoamperometry under optical modulation. (**A**) Chronoamperometric response on a Au WE to a 450-mV potential step (versus Ag/AgCl), in .5 mM K_3_[Fe(CN)_6_] + 100 mM KNO_3_ electrolyte, for on/off laser modulation. The inset shows the on/off laser modulation response measured using a photo receiver (modulation frequency of 600 Hz, period of 1.67 ms). (**B**) Zoom-in of the chronoamperometric response in the Cottrell region at the location of the blue arrow sketched in (A), resolved as eight current samples per period.

Thermally induced mass transport effects were further ruled out by taking chronoamperometry measurements, while the laser was internally modulated on/off at a frequency of 600 Hz (period of 1.67 ms). The inset in Fig. 3A shows the time response of the modulated laser power measured using a photoreceiver. The modulation frequency of 600 Hz is low enough to enable our potentiostat to reliably acquire several current samples (*8*) within a modulation period (1.67 ms), yet high enough to preclude thermally induced mass transport, which occurs on time scales of the order of seconds (*21*).

The chronoamperometric response is shown in Fig. 3A. A forward step potential of 450 mV (versus Ag/AgCl) was applied from a null potential to induce oxidation of the redox species at the Au WE, while the latter supports on/off modulated propagating SPPs. The incident laser power was modulated from 0 (off-state) to 6.3 mW (on-state), corresponding to the incident power extrema investigated in Fig. 2. The current response of Fig. 3A was plotted versus *t*^-1/2^ for the purpose of identifying the region where it decays following the Cottrell equation, indicating diffusion-limited conditions. Figure 3B plots a zoom-in of the chronoamperometric response in the Cottrell region, at the location of the blue arrow sketched in Fig. 3A, revealing a periodic response tracking in time of the laser modulation shown in the inset of Fig. 3A. The current during the laser off-state is unmeasurable (below the noise floor of our instrument), but the current during the on-state has a high signal-to-noise ratio. Recalling that thermally induced mass transport occurs over time scales of seconds rules out this process as being responsible for the increase in current.

Electrochemical impedance spectroscopy (EIS) (*33*) measurements were also performed with SPPs propagating along the Au WE, as a function of incident optical power, at λ_0_ = 1350 nm. EIS was carried out over the frequency range from 100 kHz to 1 Hz, with an AC amplitude of 5 mV root mean square (versus Ag/AgCl), under open-circuit and DC-biased (polarized) conditions (see Materials and Methods). The DC bias was set to 250 mV (versus Ag/AgCl), close to the oxidation peak observed on Fig. 2A. Nyquist plots were produced from the impedance responses, and all plots could be separated into three regions (*33*): a semicircle at high frequencies, an approximately diagonal linear trend at intermediate frequencies, and an approximately vertical linear trend at low frequencies, as can be observed in fig. S9A for the open-circuit case under no illumination.

**Fig. 4.**
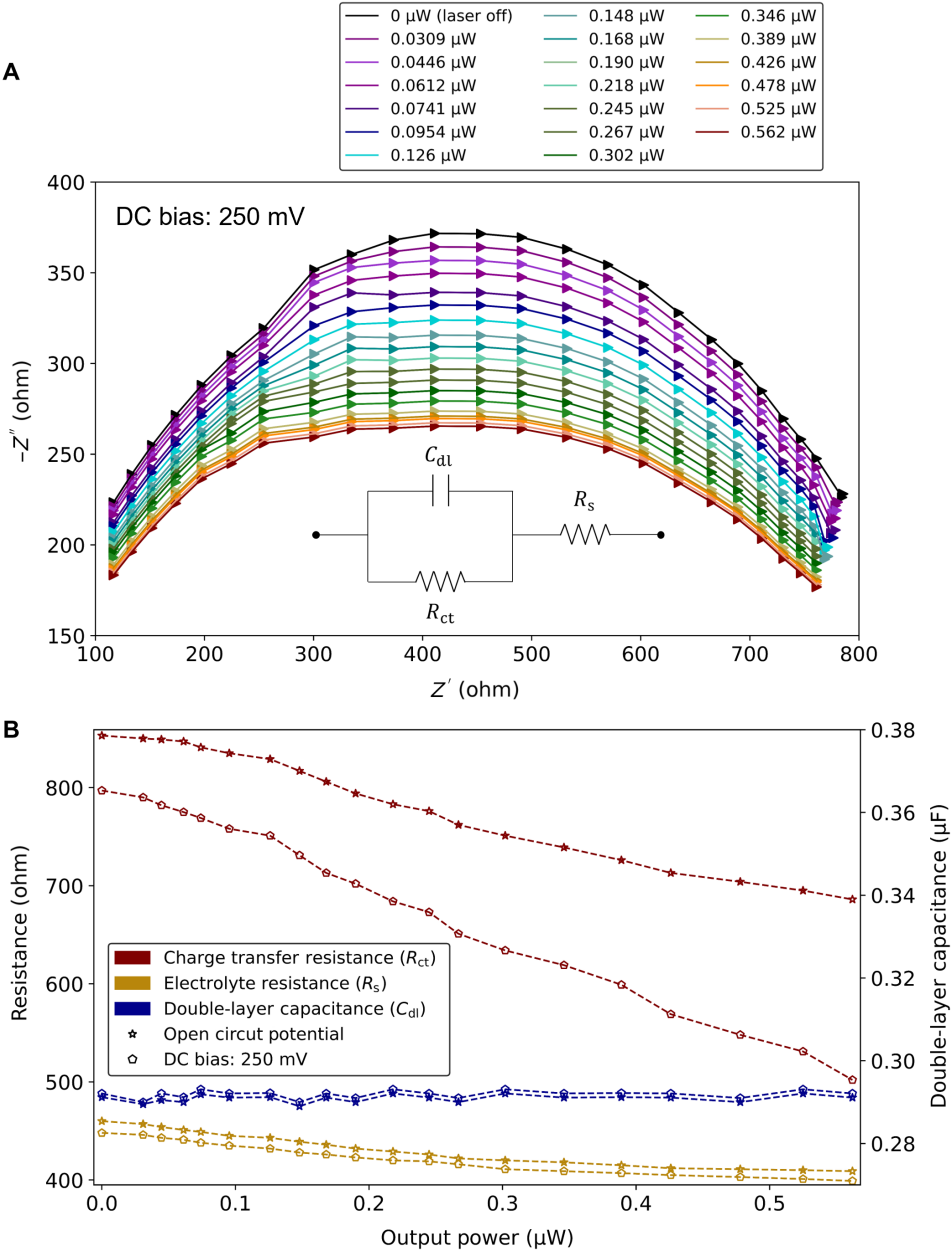
EIS under optical illumination. (**A**) Electrochemical impedance frequency responses, measured on a Au WE, in 0.5 mM K_3_[Fe(CN)_6_] + 100 mM KNO_3_ electrolyte, for increasing output optical power (legend) at λ_0_ = 1350 nm. The reference impedance response (laser off) is plotted in black. The AC potential amplitude was set to 5 mV root mean square, and the DC bias (polarization) was set to 250 mV (both versus Ag/AgCl). The impedance responses are plotted over the frequency range 100 to 3 kHz as Nyquist plots (*Z* = *Z′* + *iZ′′*). The inset shows the simplified Randle circuit composed of *C*_dl_ (double layer capacitance), *R*_ct_ (charge transfer resistance), and *R*_s_ (electrolyte resistance)*.* (**B**) Extracted Randle circuit parameters (*R*_ct_, *C*_dl_, and *R*_s_) versus output optical power under open-circuit conditions (stars) and a DC bias of 250 mV (versus Ag/AgCl) (circles).

The semicircles change significantly with optical power compared to the references (no illumination), as observed in Fig. 4A for the biased case and in fig. S9B for the open-circuit case. The impedance response of the simplified Randle equivalent circuit, shown in the inset of Fig. 4A, was fit to each semicircle of Fig. 4A and fig. S9B. The fitting frequency range was 100 to 3 kHz, which covers the range over which the semicircles are plotted. The circuit parameters of the Randle model remained unconstrained during fitting. The *R*^2^ goodness of fit was better than 0.91 over all cases. The extracted circuit parameters of the Randle model are plotted in Fig. 4B versus the output optical power. As observed, the charge transfer resistance, *R*_ct_, decreases significantly with increasing optical power for both cases and by almost 2× for the biased case. Conversely, the double layer capacitance, *C*_dl_, and the electrolyte resistance, *R*_s_, remain comparatively constant with optical power (the small decrease in *R*_s_ may be due to the small increase in WE temperature with increasing optical power; cf. fig. S4C).

## DISCUSSION

We interpret the enhancements observed in the redox currents (Figs. 2 and 3) and the drop in charge transfer resistance (Fig. 4) due to energetic carriers created along the Au WE, as SPPs propagate and are absorbed therein. Energetic electrons transfer from the WE to the redox species enhancing the reduction current, and energetic holes transfer to enhance the oxidation current—both are required to explain the results observed in Fig. 2B.

The power threshold behavior observed in Fig. 2B indicates the opening of redox channels associated with energetic carrier transfer, as these channels overcome noise in the system and the equilibrium redox currents become dominant. Beyond threshold, both trends are linear with optical power, as expected for an effect based on energetic carriers. Thermal effects on the redox currents are also linear but follow much weaker trends as noted from the measured thermal trend lines added to Fig. 2B (dashed blue). Although peak currents in CV measurements incorporate electron transfer and mass transport effects, it is clear that SPPs affect primarily electron transfer processes through the creation of energetic carriers.

The creation of energetic carriers causes the oxidation and reduction potentials to decrease significantly relative to Ag/AgCl, as observed in Fig. 2C, well beyond the thermal trend line for the equilibrium potential (dashed blue). The decrease (negative shift) to lower potentials for the oxidation reaction implies that it is easier to drive because of energetic holes, but the decrease (also a negative shift) in the reduction potential implies the opposite for energetic electrons. The difference between the peak redox potentials does not decrease with optical power as might be expected for energetic carriers of both types. This is explained by the fact that our system is electrochemically quasi-reversible (although the redox species is chemically reversible). Quasi-reversibility is evident from Fig. 2B, where the ratio of the peak redox currents is observed to differ from unity under no optical illumination, and beyond threshold as the optical power increases, i.e., the peak currents are not symmetric about zero. Nevertheless, the oxidation and reduction rates are significantly enhanced by energetic holes and electrons, as evidenced by the increased currents (Fig. 2B).

The redox potentials beyond threshold also decrease with increasing photon (SPP) energy, as excited carriers become increasingly energetic: In this region, the redox potentials are controlled optically via the photon (SPP) energy, as emphasized by the vertical blue bars in Fig. 2C.

**Fig. 5.**
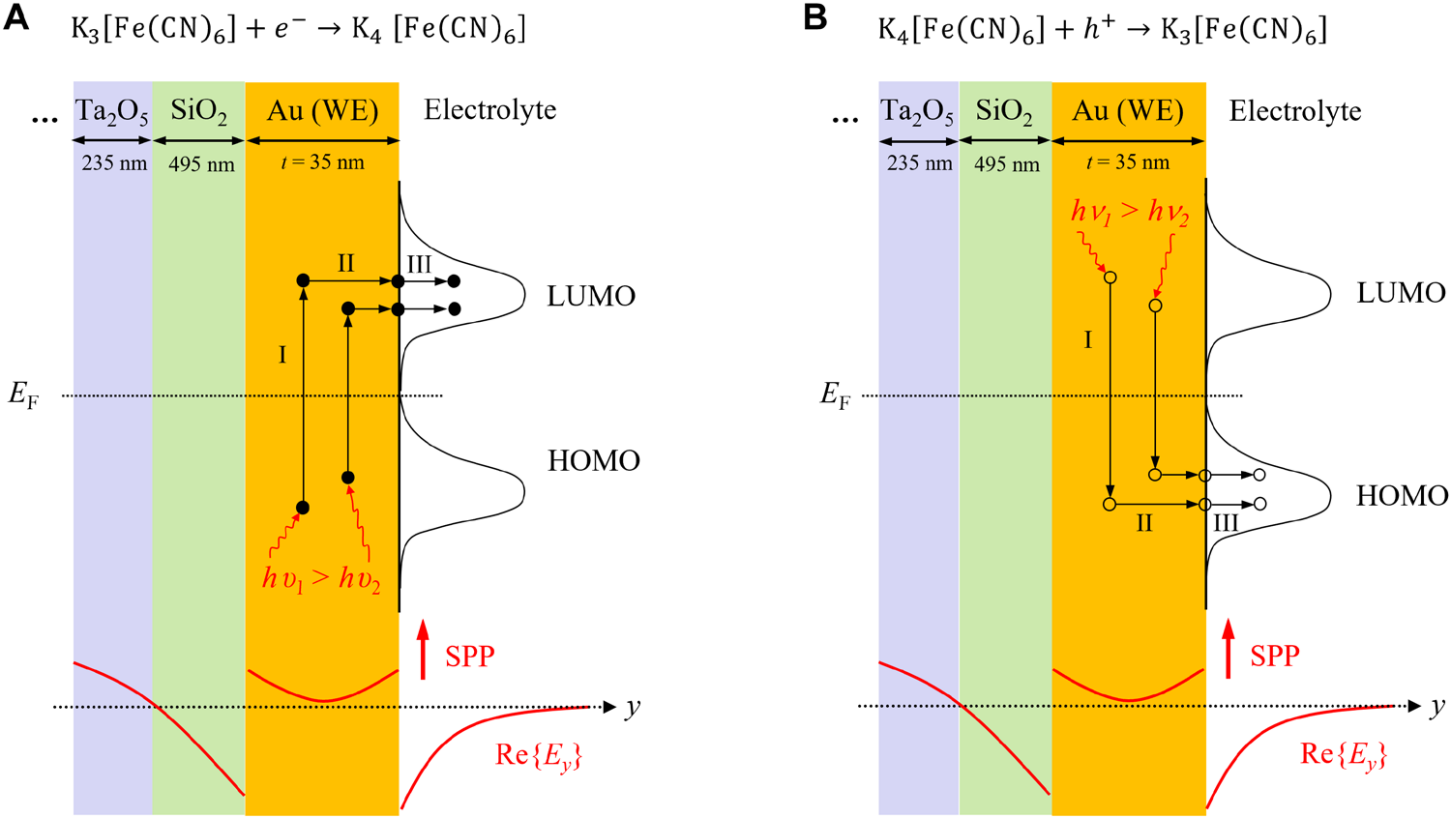
Phenomenological sketch. Propagating SPPs are absorbed in the WE where they generate energetic electrons (filled circles) and energetic holes (empty circles) (step I: photoexcitation). Some energetic electrons and holes travel to the WE surface (step II: transport), where they are emitted into LUMO or HOMO states (respectively) of the redox species (step III: emission). (**A**) Reduction reaction. (**B**) Oxidation reaction.

Figure 5 proposes a phenomenological sketch illustrating the creation and transfer of energetic carriers to the redox species as a three-step process [following internal photoemission (*26*)]. SPPs propagating along the WE are absorbed therein, leading to the photoexcitation of electrons and holes (step I). These excited carriers have excess energy that is primarily kinetic and wave vectors that are initially oriented isotropically. A fraction of the excited carriers reaches the WE surface in contact with the electrolyte (step II), and a fraction of those are emitted into states of adjacent redox molecules—electrons into LUMO (lowest unoccupied molecular orbital) states or holes into HOMO (highest occupied molecular orbital) states, depending on whether the applied potential is reductive (Fig. 5A) or oxidative (Fig. 5B).

The reduction current is enhanced to a greater extent than the oxidation current (Fig. 2). This outcome is aligned with (i) energetic electrons having a longer attenuation length than energetic holes in Au when carriers are excited within ~1 eV about *E*_F_ [*L*_e_ ~ 74 nm, *L*_h_ ~ 55 nm; (*25*)] and (ii) both types of carriers having similar energy distributions (transitions are indirect intraband near *E*_F_). A larger attenuation length increases the escape probability of electrons from the WE to the redox species, thereby producing a greater enhancement of reduction reactions.

The thickness of the WE (*t* = 35 nm) is smaller than both attenuation lengths (*L*_e_, *L*_h_), which enhances escape probabilities for both types of carriers, because energetic carriers generated throughout the WE can reach the surface in contact with the electrolyte. Furthermore, carriers that travel toward the Au/SiO_2_ interface can be reflected therefrom, eventually reaching the surface in contact with the electrolyte, further enhancing escape probabilities [cf. internal photoemission from thin films (*26*)]. This implies that thinner WEs could produce greater enhancements. Last, infrared SPPs (photons) of energy *h*υ ~ 1 eV create electrons and holes in Au that are sufficiently energetic and long-lived to significantly affect electrochemical reactions.

## MATERIALS AND METHODS

### Preparation of the waveguides and electrodes on chip

Chips incorporating Au stripes with contact pads and grating couplers and Pt stripes with contact pads were fabricated as described in previous work (*27*, *28*) but without upper cladding or lid. Chips without an upper cladding enable direct probing of pads connected to the WE and CE and facilitate alignment of the optical fibers used to couple to the gratings on chip. The upper cladding consisted of the electrolyte solution, prepared as described below. A Au stripe was used simultaneously as an SPP waveguide and WE, whereas a nearby Pt stripe was used as a CE.

The chips were cleaned manually using a swab dipped in acetone to remove protective photoresist (SPR-220) from the surface. Then, a chip was placed into a large glass vial in acetone, and the vial was placed in an ultrasonic bath for 5 min. Next, the chip was promptly rinsed with 3-4 isopropyl alcohol via pipetting and then with deionized water. Pressurized N_2_ gas was used to dry the chip. Last, the chip was placed in an ultraviolet (UV)/ozone chamber for 30 min with the UV lamp on, followed by 30 min with the UV lamp light off, as a final cleaning step.

WE and CE were then selected and electrically burned in before use by injecting a current (ramp function) along an electrode structure until its resistance stabilized. Burn-in induces grain reorganization through annealing, which stabilizes the electrodes before use in an electrochemical experiment (*34*). The chip was affixed to the bottom of a petri dish, and electrochemical, optical, or resistance measurements were carried out as required.

### Experimental setup

The experimental setup, constructed on a floating optical table, includes a tunable laser (8164A, Agilent) working over the wavelength range from 1270 to 1370 nm. A cleaved bow-tie style polarization-maintaining single-mode optical fiber (PM-SMF) with a 6.6-μm core diameter and a multimode fiber (MMF) with a core diameter of 200 μm were aligned perpendicularly to the input and output grating couplers, respectively. Metallic holders (90°) mounted on multiaxis micropositioners were used to hold the fibers while ensuring that transverse magnetic-polarized light was incident on the input grating. A power meter (PM 100USB, Thorlabs) was used to monitor the power emerging from the MMF coupled to the output grating. Following the scheme illustrated in Fig. 1B, the input PM-SMF was aligned perpendicularly to the input grating coupler such that laser light is incident thereon, exciting Bloch long-range SPPs along the Au stripe in both longitudinal directions. Propagating SPPs are outcoupled by the output grating into light captured by the MMF connected to the power meter. This arrangement in transmission enables optimization of both optical alignments and the operating wavelength.

Two tungsten needles, attached to the arms of two micropositioners, were used to probe pads at the end of a Pt stripe and a Au stripe following the scheme illustrated in Fig. 1B. The needles were coated with poly(methyl methacrylate) except for their tip to ensure no interaction with the electrolyte.

A bipotentiostat (WaveDriver 20, basic bundles, Pine Research Instrumentation) was used for the CV and chronoamperometry measurements. A potentiostat (PARSTAT 2273, Princeton Applied Research) was used for the EIS measurements, and the ZSimpWin software was used to fit the high-frequency impedance responses to the simplified Randle equivalent circuit (Fig. 4A, inset). An Ag/AgCl reference electrode (double junction pH combination, glass body, BNC connector, Sigma-Aldrich) was used in all electrochemical measurements.

The chip was immersed in electrolyte, the reference electrode was dipped nearby, and the needles were used to probe the pads of the WE and CE on chip to connect them to the bipotentiostat in a three-electrode configuration. CV, chronoamperometry, and EIS routines supplied by the manufacturers of our instruments were used to carry out the measurements.

The petri dish housing the chip and electrochemical cell was placed on a TEC. The interface between the petri dish and TEC was filled with silicon paste to ensure high thermal conductivity between these parts. A clean electronic thermometer was dipped in the electrolyte and used with a temperature controller (operating in a closed-loop feedback control algorithm) to control the temperature of the cell via the TEC, over the range from 10° to 40°C. Sufficient time was allowed to lapse whenever the temperature was changed to allow the cell and its contents (electrolyte, chip, and electrodes) to reach thermal equilibrium before any measurements were undertaken.

An electronic source meter (2400, Keithley) along with an extra pair of probe needles and micropositioners was used to measure stripe resistances. An alignment microscope of long working distance was used to align the fibers and probes to the chip. Schematics of our experimental arrangement and setup are given in fig. S1.

### Redox species and electrolyte/upper cladding

Potassium ferricyanide {K_3_[Fe(CN)_6_]} and potassium nitrate (KNO_3_) were used as supplied (Sigma-Aldrich) as the redox species and supporting electrolyte, respectively. Glycerol (Sigma-Aldrich) was used as supplied and added to the electrolyte (0.2928 g of glycerol per 20 ml of electrolyte) to adjust the refractive index of the solution because it also acts as the upper cladding of the SPP waveguide. The refractive index of the solution was adjusted to *n* = 1.3325 (λ_0_ = 1312 nm), as measured using a prism coupler (Metricon), such that the Bloch long-range SPP could propagate (*27*–*29*). CV measurements with and without glycerol were identical over the range of potentials of interest (no glycerol oxidation was observed over the potential window of 0 to 0.5 V versus Ag/AgCl used in our study).

### Mode computation

The Bloch long-range SPP mode shown in Fig. 1C was computed using the Method of Lines (*35*). The multilayer structure is composed of 15 periods of a SiO_2_ on Ta_2_O_5_ unit cell of thicknesses *d*_SiO_2__= 495 nm (*n*_SiO_2__= 1.447) and *d*_Ta_2_O_5__= 235 nm (*n*_Ta_2_O_5__= 2.069), on a starting layer of SiO_2_ of the same thickness, on a Si wafer. This multilayer was designed to achieve wave number matching at free-space wavelengths near λ_0_ = 1310 nm, as described elsewhere (*29*). The relative permittivity of the Au stripe at λ_0_ = 1310 nm was taken as ε_r_ = −86.8 − j8.322 (*36*), and its dimensions were set to *w* = 5 μm and *t* = 35 nm. The upper cladding was taken as an optically semi-infinite region of electrolyte with glycerol as described above. Figure 5 gives a partial cross-sectional sketch of the structure.

### Experimental arrangement for direct resistive heating of WE

Heating the stripe directly produces a thermal gradient in the electrolyte that resembles closely the thermal gradient produced by absorption of the propagating SPP [cf. thermal computations for comparable structures (*37*)]. The setup of fig. S7 was used to obtain CV curves, while the Au WE electrode was simultaneously heated resistively via the passage of current. A power supply (Model PAB 25-1 tr, KIKUSUI) was connected to a WE by probing the pads at each end and applying a voltage in the range of 2.5 to 8.5 V. An ammeter (Agilent U1242B multimeter) was inserted in series to measure the current *I* flowing out of the power supply. This current is of the order of milliamperes, much larger than the current flowing in/out of the WE to the redox species during CV measurements which is of the order of microamperes. The measured current *I* and the voltage applied by the power supply were used to obtain the resistance of the WE and deduce its temperature by using the temperature coefficient of resistivity of Au (fig. S4B). The voltage range was selected to increase the temperature of the WE from 20° to 50°C, in correspondence with the temperature range in our optical experiments. The temperature of the electrochemical cell was kept to 20°C using the TEC placed under the petri dish, thereby ensuring a stable thermal gradient from the WE into the electrolyte. Sufficient time was allowed to lapse whenever the voltage was changed to allow the system to reach thermal equilibrium before any measurements were recorded. The bipotentiostat was used to simultaneously obtain CV measurements on the WE, in 0.5 mM K_3_[Fe(CN)_6_] + 100 mM KNO_3_ electrolyte, at a scan rate of 100 mV/s. Interference between the instruments in the setup was not detected.

## Supplementary Materials

### This PDF file includes:

Figs. S1 to S9 References
