# Supplementary material for: Infrared surface plasmons on a Au waveguide electrode open new redox channels associated with the transfer of energetic carriers
Source: Sci Adv. 2022 May 18;8(20):eabm9303. doi: 10.1126/sciadv.abm9303 (PMC9116605; doi:10.1126/sciadv.abm9303)
Supplement: Supplementary file 1 — Figs. S1 to S9 References [file sciadv.abm9303_sm.pdf]

Science Advances
AAAS

Supplementary Materials for

Infrared surface plasmons on a Au waveguide electrode open new redox channels associated with the transfer of energetic carriers

Zohreh Hirbodvash, Oleksiy Krupin, Howard Northfield, Anthony Olivieri, Elena A. Baranova, Pierre Berini*

*Corresponding author. Email: berini@eecs.uottawa.ca

Published 18 May 2022, *Sci. Adv.* **8**, eabm9303 (2022)
DOI: 10.1126/sciadv.abm9303

This PDF file includes:

Figs. S1 to S9
References

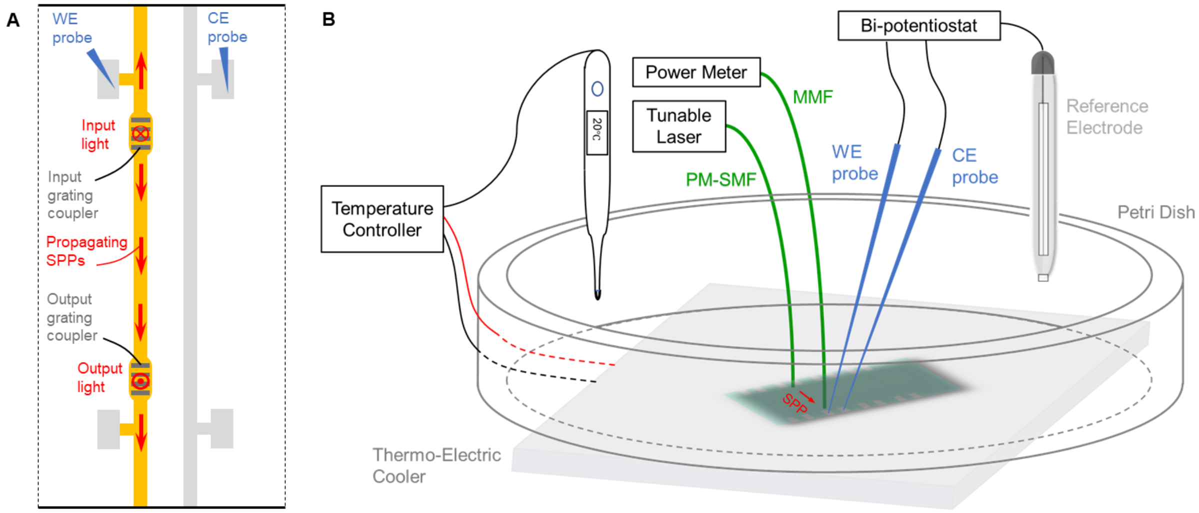

Fig. S1. Experimental set-up. (**A**) Sketch of chip and (**B**) block diagram of experimental set-up. PM-SMF: polarization-maintaining single-mode fibre; MMF: multi-mode fibre; WE: working electrode; CE: counter electrode.

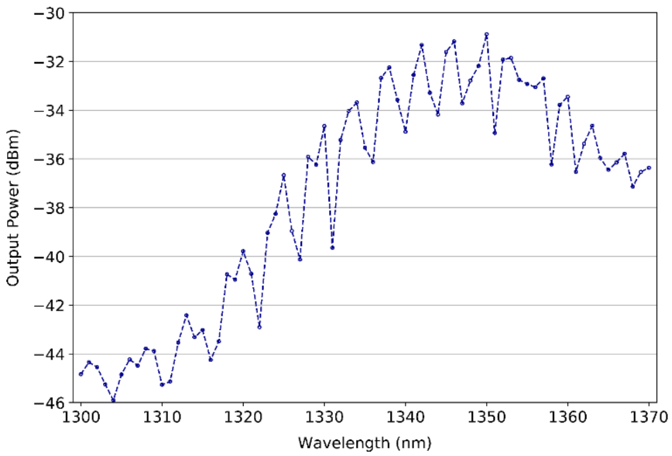

Fig. S2. Wavelength response of grating-coupled waveguide / working electrode.
Wavelength response of grating couplers, separated by a waveguide segment of length l_3 = 1850 µm (Fig. 1B, main text). The incident optical power was set to 8 dBm. The combined grating coupling losses are ~20 dB, and the propagation loss of the Bloch LRSPP along the stripe is ~12 dB/mm (*28*).

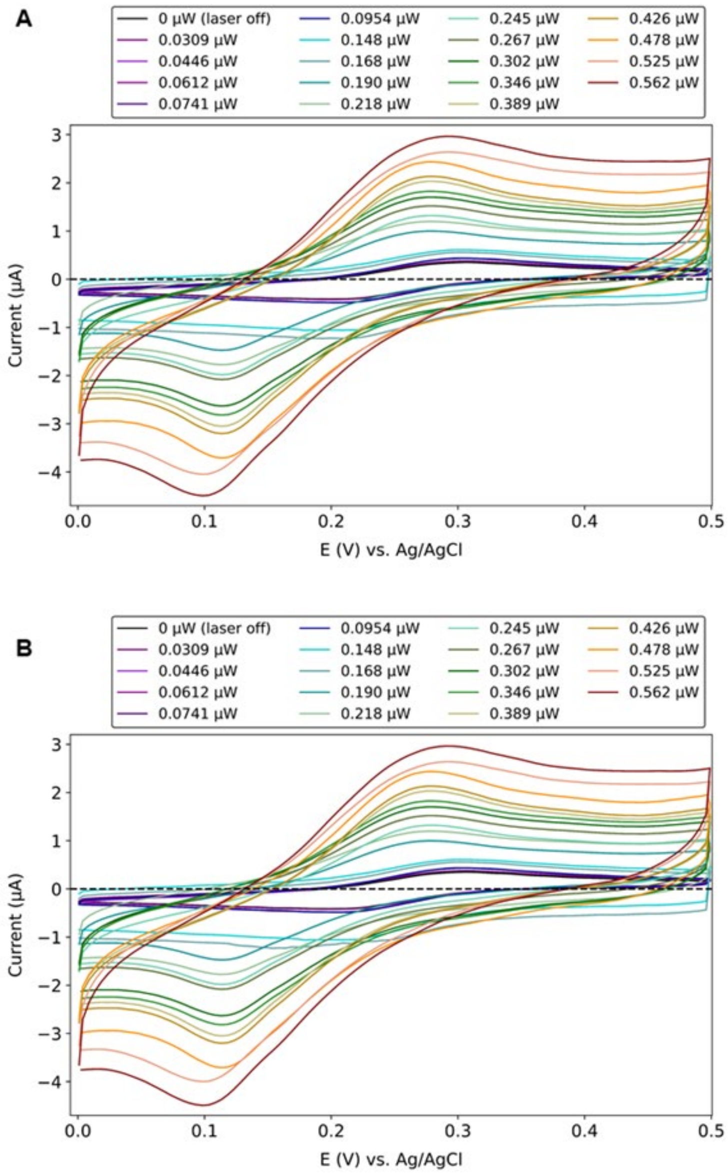

Fig. S3. Cyclic voltammetry under optical illumination. CV curves obtained on a Au WE, in 0.5 mM $K_3[Fe(CN)_6]$ + 100 mM KNO_3 electrolyte, at a scan rate of 100 mV/s, for increasing optical output power (legend) at (**A**) λ_0 = 1330 nm (**B**) λ_0 = 1370 nm. The incident optical power ranged from 0 to 6.3 mW. The reference CV curve (laser off) is plotted in black.

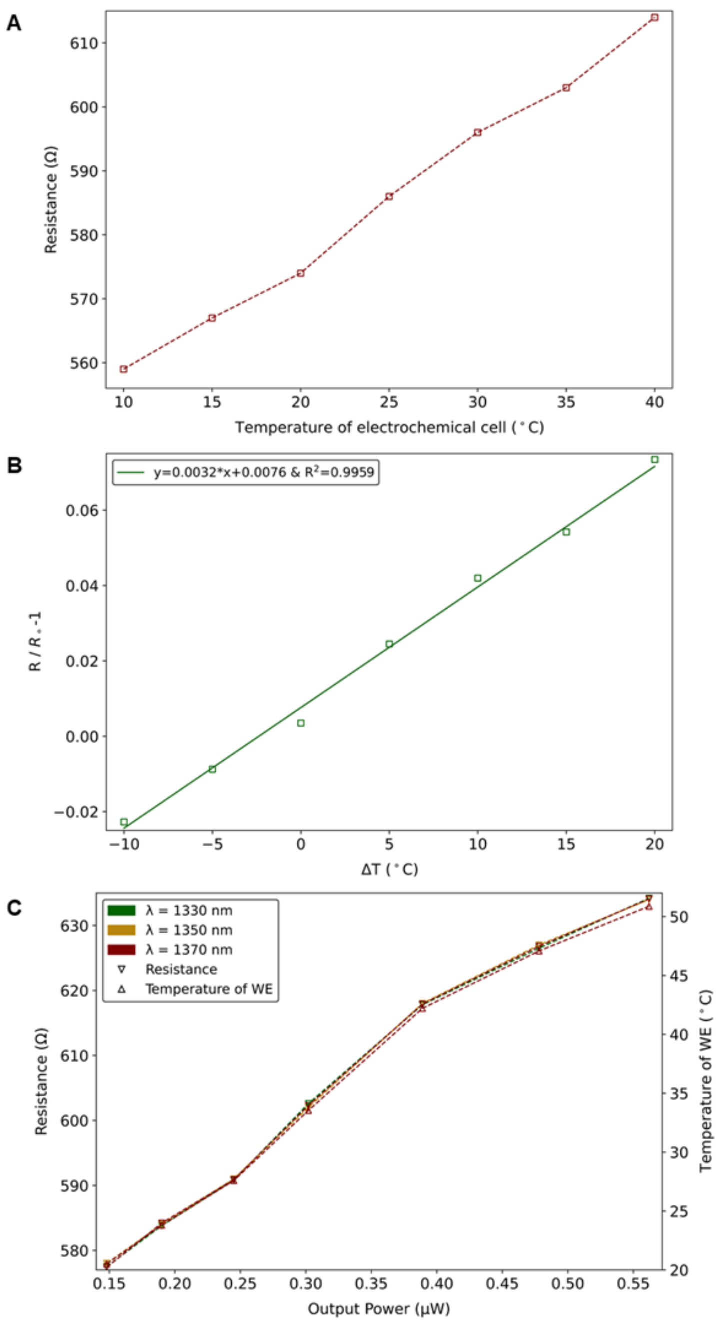

Fig. S4. Resistance of WE as a function of temperature and optical power. (**A**) Resistance of a Au WE measured *in situ vs*. the temperature of the electrochemical cell as controlled using the TEC, with the laser off. (**B**) Results of Part (A) re-plotted using $R = R_0[1+\alpha(T - T_0)]$ such that the temperature coefficient of resistivity (α) appears as the slope; R_0 is the resistance at the reference temperature of T_0 = 20 ºC, R is the resistance at temperature T, and $\Delta T = T - T_0$. The best fit linear model given in the legend yields $\alpha = 3.2\times10^{-3}$ ºC^{-1}, in excellent agreement with the literature for bulk Au (*35*). (**C**) Resistance of Au WE measured *in situ vs.* increasing output optical power (λ_0 = 1330, 1350, 1370 nm), with the electrochemical cell maintained at 20 ºC. The corresponding temperature of the WE, T, is plotted on the right axis, deduced using the relation $R = R_0[1+\alpha(T - T_0)]$ with T_0 = 20 ºC, and R_0 and α as measured in Part (B).

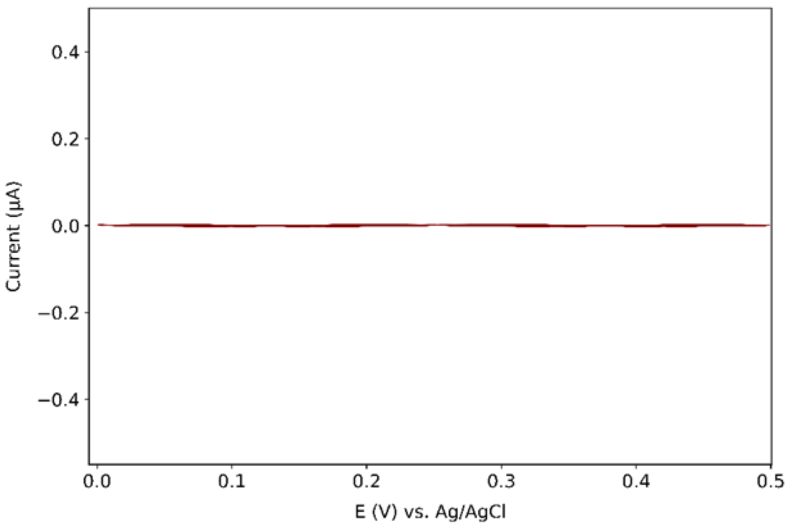

Fig. S5. CV Measurements obtained without redox species. CV curves obtained on a Au WE, in 100 mM KNO_3 electrolyte (electrolyte only), at a scan rate of 100 mV/s.

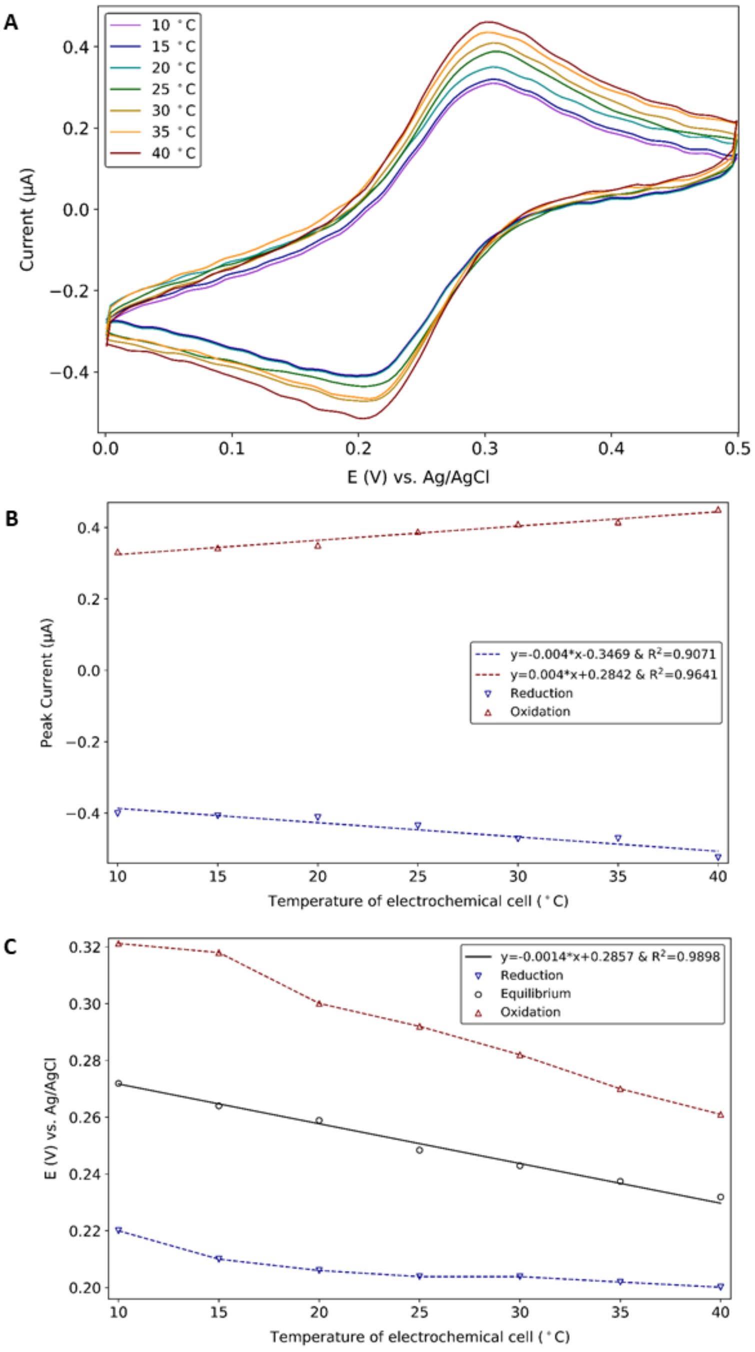

Fig. S6. Cyclic voltammetry at various cell temperatures. (**A**) CV curves obtained on a Au WE, in 0.5 mM $K_3[Fe(CN)_6]$ + 100 mM KNO_3 electrolyte, at a scan rate of 100 mV/s, as the temperature of the electrochemical cell was varied using the TEC from 10 to 40 ºC (legend). (**B**) Redox current peaks, and (**C**) potentials *vs*. cell temperature, obtained from the CV curves of Part (A). Linear models fitted to the peak redox currents are plotted as the dashed lines in Part (B) and given in the legend (slopes have units of µA/ºC). A linear model fitted to the equilibrium potential (mean of redox potentials) is plotted as the solid black line in Part (C) and given in the legend (slope has units of V/ºC). The linear models of Parts B and C are transposed to Figs. 2B and 2C of the main text (respectively) where they are plotted as dashed blue lines.

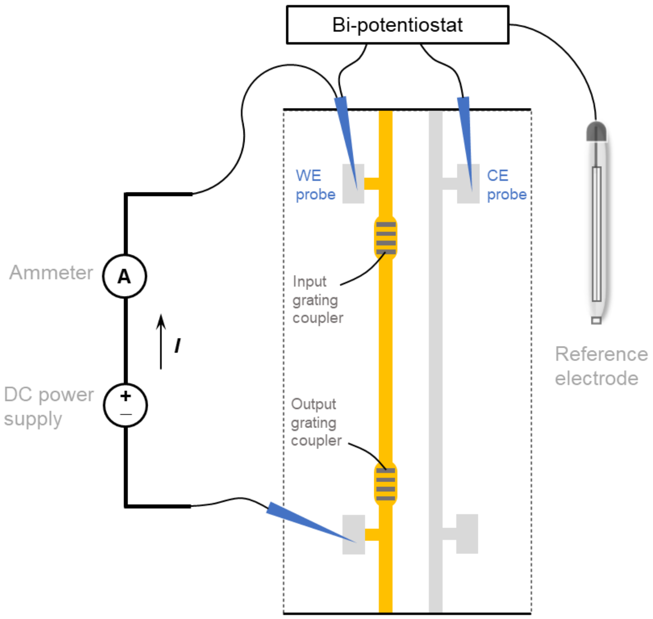

Fig. S7. Experimental arrangement for direct resistive heating of the WE. WE: working electrode; CE: counter electrode. Arrangement immersed in Petri dish on TEC as in Fig. S1B. The current *I* is measured using the ammeter and resistively heats the WE *in situ*.

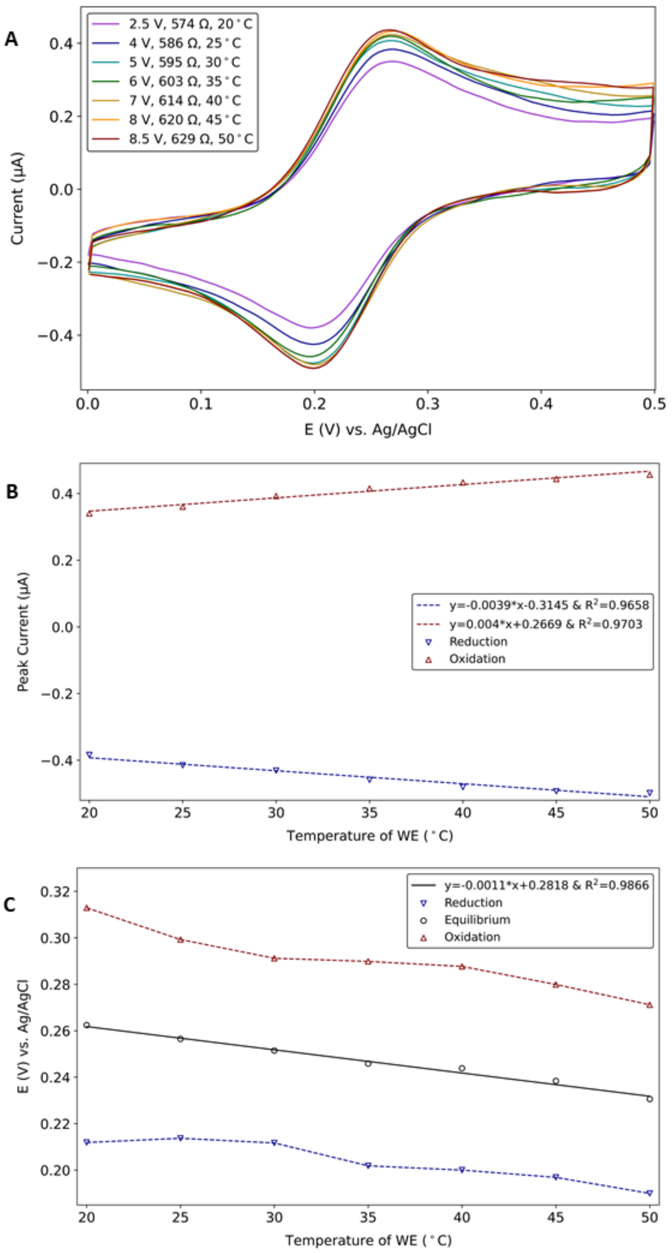

Fig. S8. Cyclic voltammetry at various WE temperatures. (**A**) CV curves obtained on a Au WE, in 0.5 mM $K_3[Fe(CN)_6]$ + 100 mM KNO_3 electrolyte, at a scan rate of 100 mV/s, as the temperature of the WE was varied from 20 to 50 ºC (legend) by direct resistive heating produced by simultaneously passing current along the WE using the experimental arrangement of Fig. S7. The temperature of the WE (legend) was deduced from its resistance using the relation $R = R_0[1+\alpha(T - T_0)]$ with α as measured in Fig. S4B. The electrochemical cell was maintained at 20 ºC using the TEC. (**B**) Redox current peaks, and (**C**) potentials *vs*. WE temperature, obtained from the CV curves of Part (A). Linear models fitted to the peak redox currents are plotted as the dashed lines in Part (B) and given in the legend (slopes have units of μA/ºC). A linear model fitted to the equilibrium potential (mean of redox potentials) is plotted as the solid black line in Part (C) and given in the legend (slope has units of V/ºC).

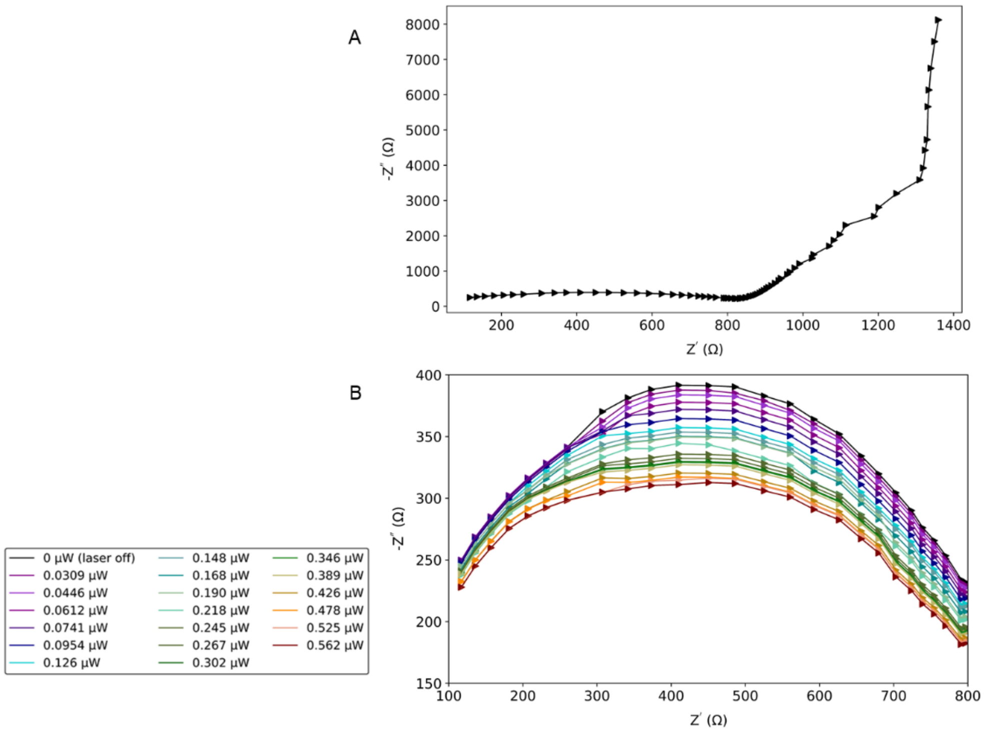

Fig. S9. Open-circuit electrochemical impedance spectroscopy. (**A**) Electrochemical impedance frequency response, measured on a Au WE, in 0.5 mM $K_3[Fe(CN)_6]$ + 100 mM KNO_3 electrolyte, under no illumination (laser off). The AC potential amplitude was set to 5 mV RMS (*vs*. Ag/AgCl). The impedance response is plotted over the frequency range 100 kHz to 1 Hz as a Nyquist plot ($Z = Z' + iZ''$). The plot comprises three regions: a semicircle at high frequencies (100 Ω < Z' < 800 Ω), an approximately diagonal linear trend at intermediate frequencies (800 < Z' < 1300 Ω), and an approximately vertical linear trend at low frequencies (Z' > 1300 Ω). The open circuit DC potential was measured as 252 mV (*vs*. Ag/AgCl) under no illumination, in agreement with the equilibrium potential observed on Fig. 2C. (**B**) High-frequency impedance responses, plotted over the frequency range 100 kHz to 3 kHz, as Nyquist plots, under optical illumination at λ_0 = 1350 nm, for increasing output optical power (legend) (other parameters are the same as in Part A). These plots were fit to the Randle equivalent circuit, and the extracted parameters (R_{ct}, C_{dl} and R_s) plotted *vs.* output optical power in Fig. 4B of the main text (stars).

REFERENCES AND NOTES

1. W. L. Barnes, A. Dereux, T. W. Ebbesen, Surface plasmon subwavelength optics. *Nature* **424**, 824–830 (2003).

2. P. Berini, Long-range surface plasmon polaritons, *Adv. Opt. Photon.* **1**, 484–588 (2009).

3. R. Sundararaman, P. Narang, A. S. Jermyn, W. A. Goddard III, H. A. Atwater, Theoretical predictions for hot-carrier generation from surface plasmon decay. *Nat. Commun.* **5**, 5788 (2014).

4. C. Clavero, Plasmon-induced hot-electron generation at nanoparticle/metal-oxide interfaces for photovoltaic and photocatalytic devices. *Nat. Photonics* **8**, 95–103 (2014).

5. M. L. Brongersma, N. J. Halas, P. Nordlander, Plasmon-induced hot carrier science and technology. *Nat. Nanotechnol.* **10**, 25–34 (2015).

6. S. Linic, U. Aslam, C. Boerigter, M. Morabito, Photochemical transformations on plasmonic metal nanoparticles. *Nat. Mater.* **14**, 567–576 (2015).

7. Y. Zhang, S. He, W. Guo, Y. Hu, J. Huang, J. R. Mulcahy, W. D. Wei, Surface-plasmon-driven hot electron photochemistry. *Chem. Rev.* **118**, 2927–2954 (2018).

8. X. Shan, U. Patel, S. Wang, R. Iglesias, N. Tao, Imaging local electrochemical current via surface plasmon resonance. *Science* **327**, 1363–1366 (2010).

9. S. Wang, X. Huang, X. Shan, K. J. Foley, N. Tao, Electrochemical surface plasmon resonance: Basic formalism and experimental validation. *Anal. Chem.* **82**, 935–941 (2010).

10. T. Sannomiya, H. Dermutz, C. Hafner, J. Vörös, A. B. Dahlin, Electrochemistry on a localized surface plasmon resonance sensor. *Langmuir* **26**, 7619–7626 (2010).

11. C. Novo, A. M. Funston, A. K. Gooding, P. Mulvaney, Electrochemical charging of single gold nanorods. *J. Am. Chem. Soc.* **131**, 14664–14666 (2009).

12. A. B. Dahlin, B. Dielacher, P. Rajendran, K. Sugihara, T. Sannomiya, M. Zenobi-Wong, J. Vörös, Electrochemical plasmonic sensors. *Anal. Bioanal. Chem.* **402**, 1773–1784 (2012).

13. Y. Zhang, W. Guo, Y. Zhang, W. D. Wei, Plasmonic photoelectrochemistry: In view of hot carriers. *Adv. Mater.* **33**, 2006654 (2021).

14. L. Zhou, D. F. Swearer, C. Zhang, H. Robatjazi, H. Zhao, L. Henderson, L. Dong, P. Christopher, E. A. Carter, P. Nordlander, N. J. Halas, Quantifying hot carrier and thermal contributions in plasmonic photocatalysis. *Science* **362**, 69–72 (2018).

15. P. K. Jain, Taking the heat off of plasmonic chemistry. *J. Phys. Chem. C* **123**, 24347–24351 (2019).

16. G. Baffou, I. Bordacchini, A. Baldi, R. Quidant, Simple experimental procedures to distinguish photothermal from hot-carrier processes in plasmonics. *Light Sci. Appl.* **9**, 108 (2020).

17. E. Cortés, L. V. Besteiro, A. Alabastri, A. Baldi, G. Tagliabue, A. Demetriadou, P. Narang, Challenges in plasmonic catalysis. *ACS Nano* **14**, 16202–16219 (2020).

18. Y. Dubi, I. W. Un, Y. Sivan, Thermal effects – An alternative mechanism for plasmon-assisted photocatalysis. *Chem. Sci.* **11**, 5017–5027 (2020).

19. Y. Yu, J. D. Williams, K. A. Willets, Quantifying photothermal heating at plasmonic nanoparticles by scanning electrochemical microscopy. *Faraday Discuss.* **210**, 29–39 (2018).

20. Y. Yu, V. Sundaresan, K. A. Willets, Hot carriers versus thermal effects: Resolving the enhancement mechanisms for plasmon-mediated photoelectrochemical reactions. *J. Phys. Chem. C* **122**, 5040–5048 (2018).

21. M. Maley, J. W. Hill, P. Saha, J. D. Walmsley, C. M. Hill, The role of heating in the electrochemical response of plasmonic nanostructures under illumination. *J. Phys. Chem. C* **123**, 12390–12399 (2019).

22. C. Zhan, B.-W. Liu, Y.-F. Huang, S. Hu, B. Ren, M. Moskovits, Z.-Q. Tian, Disentangling charge carrier from photothermal effects in plasmonic metal nanostructures. *Nat. Commun.* **10**, 2671 (2019).

23. A. J. Wilson, V. Mohan, P. K. Jain, Mechanistic understanding of plasmon-enhanced electrochemistry. *J. Phys. Chem. C* **123**, 29360–29369 (2019).

24. M. Bauer, A. Marienfeld, M. Aeschlimann, Hot electron lifetimes in metals probed by time-resolved two-photon photoemission. *Prog. Surf. Sci.* **90**, 319–376 (2015).

25. R. N. Stuart, F. Wooten, W. E. Spicer, Mean free path of hot electrons and holes in metals. *Phys. Rev. Lett.* **10**, 7–9 (1963).

26. C. Scales, P. Berini, Thin-film Schottky barrier photodetector models. *IEEE J. Quant. Electr.* **46**, 633–643 (2010).

27. N. R. Fong, M. Menotti, E. Lisicka-Skrzek, H. Northfield, A. Olivieri, N. Tait, M. Liscidini, P. Berini, Bloch long-range surface plasmon polaritons on metal stripe waveguides on a multi-layer substrate. *ACS Photonics* **4**, 593–599 (2017).

28. M. Khodami, Z. Hirbodvash, O. Krupin, W. R. Wong, E. Lisicka-Skrzek, H. Northfield, C. Hahn, P. Berini, Fabrication of Bloch long range surface plasmon waveguides integrating counter electrodes and microfluidic channels for multimodal biosensing. *J. Microelectromech. Syst.* **30**, 686–695 (2021).

29. M. Khodami, P. Berini, Grating couplers for (Bloch) long-range surface plasmons on metal stripe waveguides. *J. Opt. Soc. Am. B* **36**, 1921–1930 (2019).

30. U. K. Sur, F. Marken, N. Rees, B. A. Coles, R. G. Compton, R. Seager, Microwave enhanced electrochemistry: Mass transport effects and steady state voltammetry in the sub-millisecond time domain. *J. Electroanal. Chem.* **573**, 175–182 (2004).

31. J. E. O'Reilly, Oxidation-reduction potential of the ferro-ferricyanide system in buffer solutions. *Biochim. Biophys. Acta* **292**, 509–515 (1973).

32. T. Kim, J. S. Lee, G. Lee, H. Yoon, J. Yoon, T. J. Kang, Y. H. Kim, High thermopower of ferri/ferrocyanide redox couple in organic-water solutions. Nano Energy **31**, 160–167 (2017).

33. B.-A. Mei, O. Munteshari, J. Lau, B. Dunn, L. Pilon, Physical interpretations of nyquist plots for EDLC electrodes and devices. *J. Phys. Chem. C* **122**, 194–206 (2018).

34. Z. Hirbodvash, M. S. E. Houache, O. Krupin, M. Khodami, H. Northfield, A. Olivieri, E. A. Baranova, P. Berini, Electrochemical performance of lithographically-defined micro-electrodes for integration and device applications. *Chem* **9**, 277 (2021).

35. P. Berini, K. Wu, Modeling lossy anisotropic dielectric waveguides with the method of lines. *IEEE Trans. Microw. Theory Techn.* **44**, 749–759 (1996).

36. E. D. Palik, Ed., *Handbook of Optical Constants of Solids* (Academic Press, 1985).

37. G. Gagnon, N. Lahoud, G. A. Mattiussi, P. Berini, Thermally activated variable attenuation of long-range surface plasmon-polariton waves. *J. Lightw. Technol.* **24**, 4391–4402 (2006).

38. R. B. Belser, W. H. Hicklin, Temperature coefficients of resistance of metallic films in the temperature range 25° to 600 °C. *J. Appl. Phys.* **30**, 313–322 (1959).
